# Supplementary material for: Value of Glycemic Indices for Delayed Cerebral Ischemia after Aneurysmal Subarachnoid Hemorrhage: A Retrospective Single-Center Study
Source: Brain Sci. 2024 Aug 23;14(9):849. doi: 10.3390/brainsci14090849 (PMC11430037; doi:10.3390/brainsci14090849)
Supplement: Supplementary file 1 [file brainsci-14-00849-s001.zip › brainsci-3154882-supplementary.pdf]

Supplementary Material

Value of Glycemic Indices for Delayed Cerebral Ischemia after Aneurysmal Subarachnoid Hemorrhage: A Retrospective Single-Center Study

Matthias Manfred Deininger [1,*], Miriam Weiss [2,3], Stephanie Wied [4], Alexandra Schlycht [1], Nico Haehn [1], Gernot Marx [1], Anke Hoellig [2], Gerrit Alexander Schubert [2,3] and Thomas Breuer [1]

[1] Department of Intensive and Intermediate Care, Medical Faculty, RWTH Aachen University, 52074 Aachen, Germany; aschlycht@ukaachen.de (A.S.); nhaehn@ukaachen.de (N.H.); gmarx@ukaachen.de (G.M.); tbreuer@ukaachen.de (T.B.)
[2] Department of Neurosurgery, Medical Faculty, RWTH Aachen University, 52074 Aachen, Germany; miriam_weiss@icloud.com (M.W.); ahoellig@ukaachen.de (A.H.); gerrit.schubert@ksa.ch (G.A.S.)
[3] Department of Neurosurgery, Cantonal Hospital Aarau, 5001 Aarau, Switzerland
[4] Institute of Medical Statistics, RWTH Aachen University, 52074 Aachen, Germany
* Correspondence: mdeininger@ukaachen.de

Table S1. Univariable logistic regression for patient characteristics and glucose indices

Results of univariable logistic regression are presented as odds ratio (OR) and 95% confidence interval (95%-CI). Significant p-values are in bold.
AACTD: Average absolute change by time difference, TWAG: Time-weighted average glucose

Variable	OR	95%-CI	p-value
Modified Fisher score	3.967	2.006 to 7.843	**<0.001**
Hunt and Hess score	1.587	0.791 to 3.187	0.194
Age	0.972	0.946 to 0.998	**0.039**
Sex (female)	1.153	0.583 to 2.278	0.683
TWAG	1.020	1.002 to 1.039	**0.029**
AACTD	1.092	0.945 to 1.263	0.233

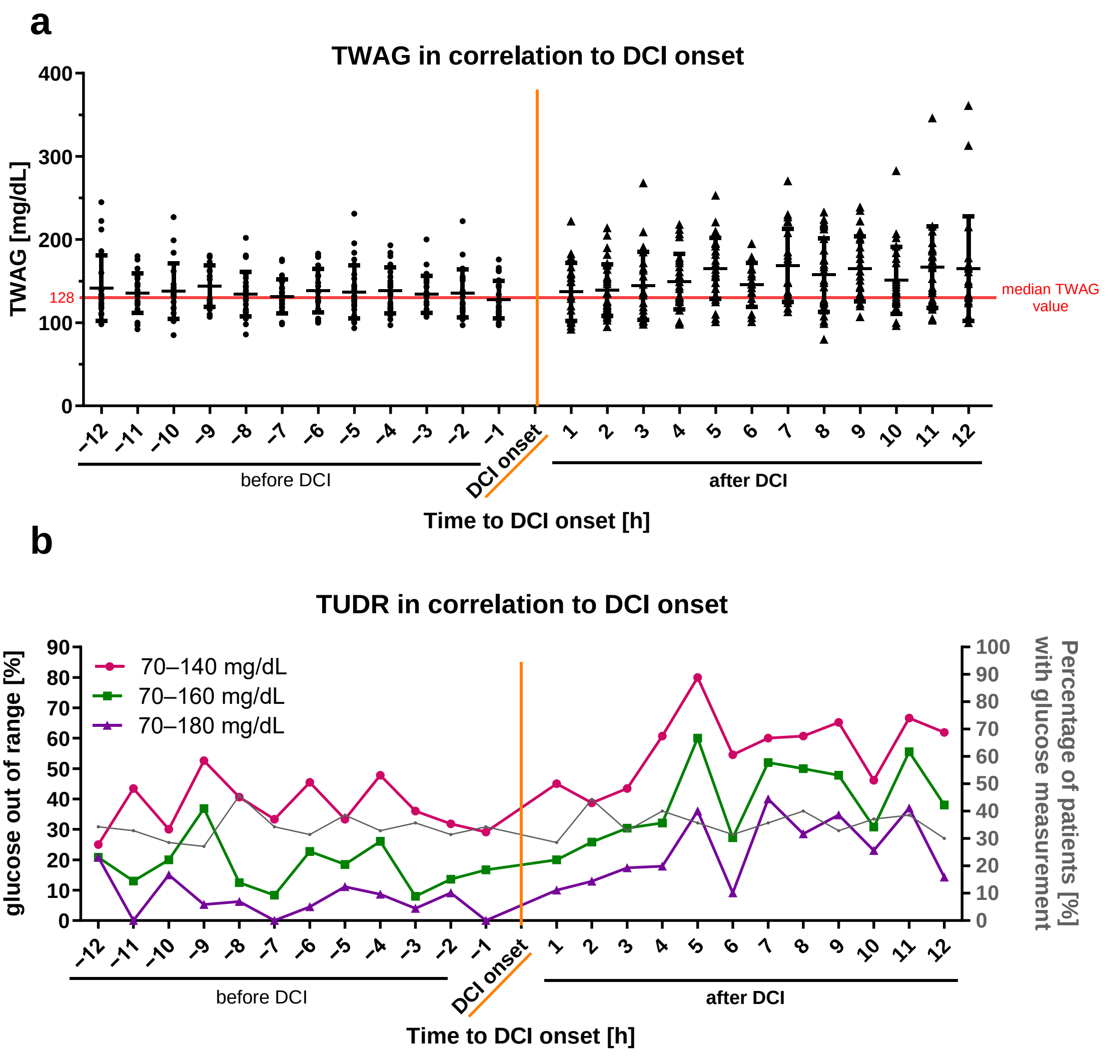

Figure S1. Glucose profile time-series for DCI patients before and after DCI-onset (1h-period).

(**a**) Cumulated time-weighted average glucose (TWAG) and (**b**) time-unified dysglycemic rate (TUDR) for every hour of aSAH patients suffering from DCI are plotted in relation to the DCI-onset (time-point zero). Periods before DCI onset are defined as negative, after the onset as positive. Dysglycemic rates are illustrated with cutoffs of <70 mg/dL and >140 mg/dL (pink color), >160 mg/dL (green color) or >180 mg/dL (violet color). Dysglycemic rate is shown as percentage of patients with at least one blood glucose measurement out of the glucose target range relative to the total number of patients with measurements in the respective 1h-period. The number of patients with at least one measurement in the respective period is plotted in percentage to total patient number on the right ordinate in grey color in (b). Blood glucose values are plotted separately for every patient. TWAG mean ± SD is shown as horizontal lines. The overall median TWAG value pre-DCI (128.7 mg/dL) is shown as a red horizontal line for orientation in (a). Due to the small number of patients with measurements per hour (34.7±4.7%) and the consequent non-representativeness of the data for the study cohort, no statistical analysis was performed.

aSAH: Aneurysmal subarachnoid hemorrhage, DCI: Delayed cerebral ischemia, ICU: Intensive care unit, TUDR: Time-unified dysglycemic rate, TWAG: Time-weighted average glucose
